# COVID-19 infection: the China and Italy perspectives

**DOI:** 10.1038/s41419-020-2603-0

**Published:** 2020-06-08

**Authors:** Jun Chen, Hongzhou Lu, Gerry Melino, Stefania Boccia, Mauro Piacentini, Walter Ricciardi, Ying Wang, Yufang Shi, Tongyu Zhu

**Affiliations:** 1 Shanghai Public Health Clinical Center, Shanghai, 201508 China; 2 TOR, University of Rome Tor Vergata, 00133 Rome, Italy; 3 Department of Health Sciences and Public Health, Università Cattolica del Sacro Cuore, Rome, Italy; 4 Department of Woman and Child Health and Public Health, Fondazione Policlinico Universitario A. Gemelli, Rome, Italy; 5 National Institute for Infectious Diseases IRCCS “Lazzaro Spallanzani”, Rome, Italy; 6 Shanghai Institute of Nutrition and Health, Shanghai Institutes for Biological Sciences, Chinese Academy of Sciences, 320 Yueyang Road, Shanghai, 200031 China; 7 The First Affiliated Hospital of Soochow University, State Key Laboratory of Radiation Medicine and Protection, Institutes for Translational Medicine, Soochow University Medical College, Suzhou, China

**Keywords:** Viral infection, Respiratory tract diseases

## Abstract

The severe acute respiratory syndrome coronavirus 2 (SARS-CoV-2) is responsible for the COVID-19 pandemic. Since its first report in December 2019, despite great efforts made in almost every country worldwide, this disease continues to spread globally, especially in most parts of Europe, Iran, and the United States. Here, we update the recent understanding in clinical characteristics, diagnosis strategies, as well as clinical management of COVID-19 in China as compared to Italy, with the purpose to integrate the China experience with the global efforts to outline references for prevention, basic research, treatment as well as final control of the disease. Being the first two countries we feel appropriate to evaluate the evolution of the disease as well as the early result of the treatment, in order to offer a different baseline to other countries. It is also interesting to compare two countries, with a very significant difference in population, where the morbidity and mortality has been so different, and unrelated to the size of the country.

## Facts

- The Covid-19 pandemic exploded first in China and subsequently in Italy. We therefore compared these two countries with the earliest and strongest impact on the population.
- With over 5,525,245 cases, including 2,315,909 recovered and 347,108 deaths worldwide as of 25 May 2020, the spread of infection still seems difficult to contain.
- Both the availability of intensive care and the local medical heath system on the territory may be responsible for the vast heterogeneity in infection containment and mortality.

## Open questions

- The therapy, especially in the advanced stages, seems complicated, and we are waiting the outcome of several hundreds of clinical trials, often duplicated and non-coordinated. Major drugs are mostly non-viral-specific: hydroxychloroquine, lopinavir/ritonavir, remdesivir, convalescent plasma/monoclonal antibody, camostat, and ivermectin. New drug development is clearly the first priority.
- The role of immunity is crucial both to overcame the acute phase and for establishing the vaccine.
- Exit from the acute phase and establishing the new protocol for the post-acute phase is of utter priority.

The severe acute respiratory syndrome coronavirus 2 (SARS-CoV-2) is responsible for the COVID-19 disease as originally shown in Wuhan, China, as early as documented from 1 December 2019 (ref. ^1^). On 11 March 2020, the WHO announced COVID-19 as a worldwide pandemic, 2 months after the official disclosure from the Chinese government of the actual cluster epidemics in Whuan^2^.

**Fig. 1:**
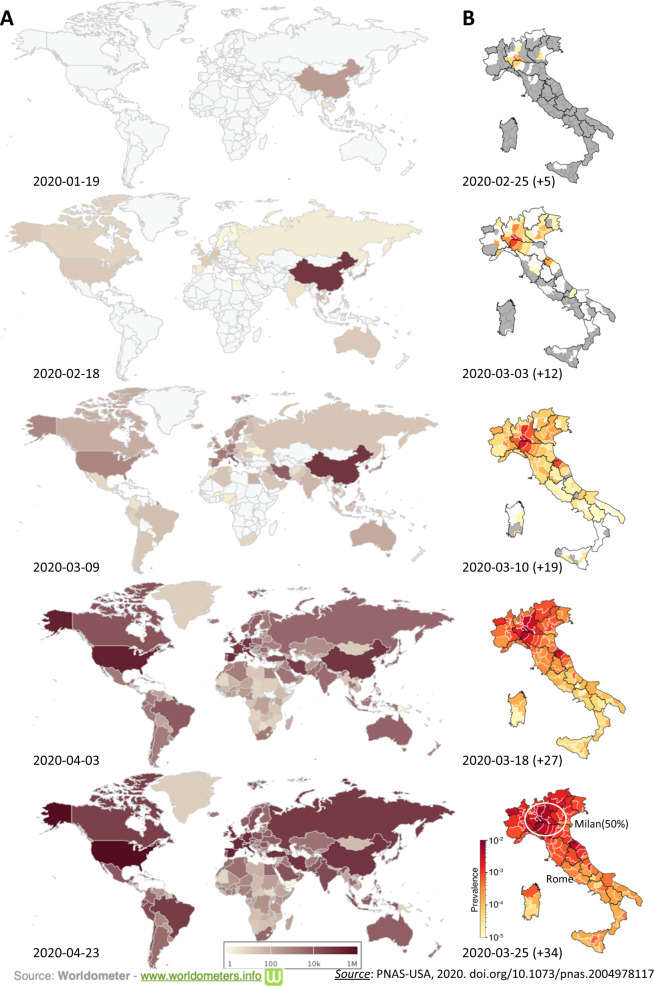
Spread and dynamics of the COVID-19 pandemic. Spread and dynamics of the COVID-19 pandemic on a worldwide scale (**a**), source Worldmeter (www.worldmeters.info), and on Italy (**b**), modified with permission from *PNAS USA*, 2020. 10.1073/pnas.2004978117.

**Fig. 2:**
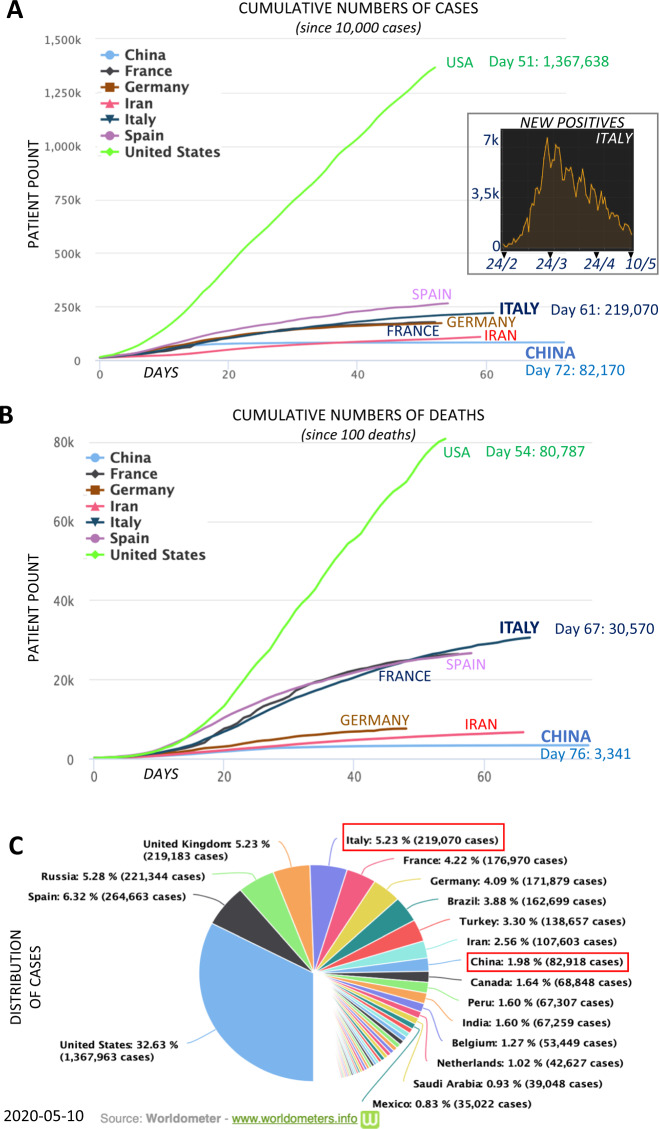
COVID-19 pandemic in different countries. Cumulative numbers of cases (**a**) and deaths (**b**) in China, European, and the United States. Distributions of the cases (**c**). The inset in panel **a** reports the new cases in Italy as to 10 May, showing a drastic reduction on morbidity. Source Worldmeter (www.worldmeters.info) and Protezione Civile (http://www.protezionecivile.gov.it/attivita-rischi/rischio-sanitario/emergenze/coronavirus). Distribution of laboratory confirmed cases of COVID-19 in the Italy, China, and the USA, as of 10 May 2020.

As of 25 May 2020, there were over 5,525,245 confirmed COVID-19 cases worldwide, of which more than 30% cases in the EU and UK, with more than 347,108 deaths worldwide^3^. Figures 1 and 2 show the current set of data of the pandemic, with a special attention to China and Italy. With the great efforts mostly based on strict containment measures, China has successfully emerged out of the first wave of the epidemic at the beginning of April 2020, when the social order slowly returned to the norms. Although different precautionary measures have been taken at national level in the EU to limit and to monitor the entrance of potential COVID-19 cases from China, the first symptomatic cases have been reported before the end of February in a number of EU countries including Italy, Spain, Germany, and, just weeks later, UK. In this context, Italy is one of the first and hardest hit country in Europe, with over 219,000 cases and 30,500 deaths reported.

**Fig. 3:**
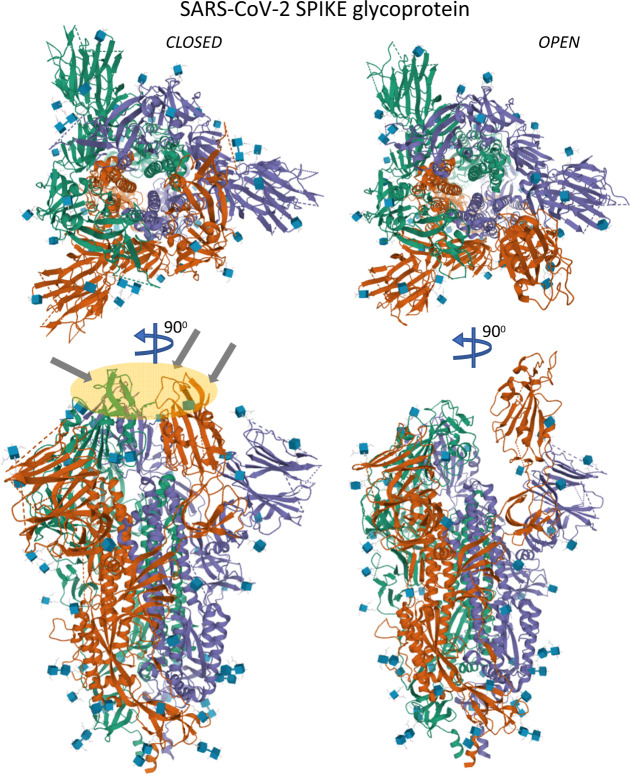
SARS-Cov spike glycoprotein. The coronavirus SARS-Cov-2 spike (S) glycoprotein is responsible for viral entry into the cell and it is the major target of antibody recognition. SARS-Cov-2 S is an ectodomain trimer. All three coronaviruses causing severe acuter respiratory syndrome coronavirus (SARS-Cov), Middle-East respiratory syndrome coronavirus (MERS-CoV), and SARS-CoV-2 are closely related and contain a transmembrane spike, S, glycoprotein with two functional subunits able to bind the cells (S1 subunit) and responsible for fusion of the viral and cellular membrane (S2 subunit). SARS-Cov-2 spike S shows a closed and an open conformation (depicted), where only the closed conformation binds the human ACE2 receptor-binding domain. The structure shown was obtained by Cryo-EM (closed: PBD = 6VXX at 2.8 Å resolution; open: PBD = 6VYB at 3.2 Å resolution) was released on 11 March 2020 from Walls et al.^4^.

**Fig. 4:**
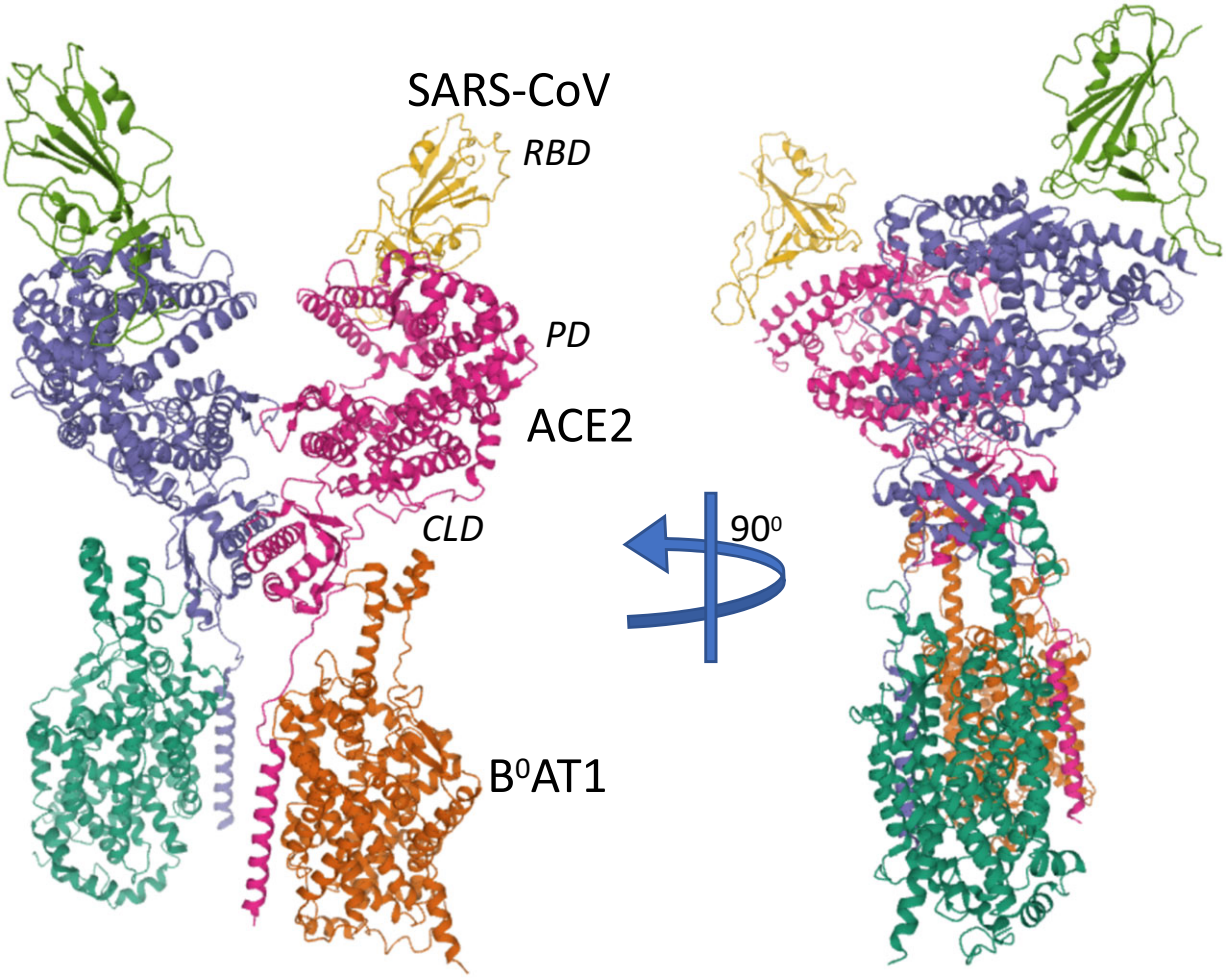
ACE2 receptor binding the SARS-Cov-2 virus. The initial step of SARS-Cov-2 viral entry during the infection is the binding of the viral trimeric spike protein (cleaved into S1 and S2 subunits, the former of which contains the receptor-binding domain, RBD) to the dimeric human receptor angiotensin-converting enzyme 2 (ACE2) which is here represented in the complex with the membrane protein that it chaperones, BoAT1. ACE2 is formed by an N-terminal peptidase domain (PD) and the C-terminal collectrin-like domain (CLD). ACE2 shows a closed and an open (depicted) conformation at the PD level of contact; however, only the closed conformation binds the RBD of SARS-Cov-2. The structure shown was obtained by Cryo-EM at 2.9 Å resolution (PBD = 6M17) was released on 11 March 2020 from Yan et al.^6^.

The SARS-CoV-2 virus is structurally and functionally closely related to the other coronaviruses causing Middle-East respiratory syndrome coronavirus (MERS-CoV) and severe acute respiratory syndrome coronavirus (SARS-CoV). In particular, it is the external protein of the virus, the glycoprotein spike (S) (Fig. 3), that is able to bind and recognize the human receptor angiotensin-converting enzyme 2 (ACE2), primed by the transmembrane protease serine type 2 (TMPRSS2), and/or CD147 (extracellular matrix metalloproteinase inducer), and therefore infect the host cells^4–6^ (Fig. 4). The crucial recent elucidation of these structures at the fine biochemical level will pave the way for future potential therapeutic interventions. The viral genomic sequences assembled from samples in Shanghai together with uploaded sequences in the Global Initiative on Sharing All Influenza Data (GISAID, www.gisaid.org) also showed a stable evolution (H.L., unpublished data). Based on current data, SARS-CoV-2 seems to have a mutation rate of around 25 mutations per year, which is much lower than that of influenza virus^7^. Therefore, development of effective long-lasting vaccines against the virus is possible.

In this review, we update the latest clinical characteristics, diagnosis strategies, as well as clinical management of the COVID-19 in China. We decided to compare the data of China and Italy because these were the first two countries hit by the pandemic at the highest level; therefore, this experience may be of relevance for other countries. Being the first two countries we feel appropriate to evaluate the evolution of the disease as well as the early result of the treatment, in order to offer a different baseline to other countries. It is also interesting to compare two countries, with a very significant difference in population, where the morbidity and mortality has been so different and unrelated to the size of the country.

## Clinical and laboratory characteristics

The COVID-19 clinical features in adults are reported in Table 1. The median incubation period (interquartile range, 2–7) is around 4 days^8^. Fever is one of the most common symptoms occurring in as high as 98% of the patients reported^1^. A relatively high proportion of patients develop fever although they are afebrile at the onset of the disease^8^. The predominant symptom is dry cough, reported in around 60% of the patients^8^. Gastrointestinal symptoms including nausea, vomiting, and diarrhea are less frequently developed, with an incidence lower than 10% of the patients^1,8,9^. Other symptoms including rhinorrhoea, sore throat, fatigue, dyspnea, muscle weakness, dizziness, and headache are also often reported in COVID-19 patients.

**Table 1:** Comparison of clinical data in china versus Italy.

		China^a^ (*N* = 44,672)	China^b^ (*N* = 113 deaths)	Italy^c^ (*N* = 127,790)	Italy^d^ (*N* = 1591 ICU patients)	Italy^e^ (*N* = 14,860 deaths)
	Age (median, interquartile range)	N/A	68 (62–77)	62 (IQR, N/A)	63 (56–70)	80 (73–85)
	*Age (group)*					
	0–18	965 (2.2%)	N/A	2045 (1.6%)	N/A	1(0)
	19–50	19,970 (44.7%)	N/A	46,132 (36.1%)	N/A	177 (1.1%)
	51–70	18,591 (41.6%)	N/A	45,493 (35.6%)	N/A	2291 (15.4)
	>70	5326 (11.9%)	N/A	34,120 (26.7%)	N/A	12,390 (83.4%)
	*Gender*					
	Male	22,981(51.4%)	83 (73%)	67,601 (52.9%)	1304 (82%)	10,062 (67.7%)
	Female	21691 (48.6%)	30 (27%)	60,189 (47.1%)	287 (18%)	4798 (32.3%)
	*Symptoms*					
	Asymptomatic	36160 (80.9%)	N/A	4169 (8.5%)	N/A	N/A
	Paucisymptomatic		N/A	7945 (16.2%)	N/A	N/A
	Mild symptoms		N/A	18,832 (38.4%)	N/A	N/A
	Severe symptoms	6168 (13.8%)	N/A	9416 (19.2%)	N/A	N/A
	Critical	2087 (4.7%)	N/A	1324 (2.7%)	N/A	N/A
	Not specified	257 (0.6%)	N/A	7356 (15.0%)	N/A	N/A
	*Sign symptoms*					
	Fever	N/A	104 (92%)	N/A	N/A	76.0%^f^
	Dyspnea	N/A	70 (62%)	N/A	N/A	72%^f^
	Cough	N/A	79 (70%)	N/A	N/A	39%^f^
	Fatigue	N/A	64 (57%)	N/A	N/A	N/A
	Diarrhea	N/A	27 (24%)	N/A	N/A	6%^f^
	Dizziness and headache	N/A	21 (19%)	N/A	N/A	N/A
	*Co-morbidity*					
	Cardiovascular and cerebrovascular diseases	3556 (17.0%)	70 (61.9%)	N/A	223 (21%) (hypertension 509 (49%)	Ischemic heart disease: 363 (28.1%); atrial fibrillation: 290 (22.5%); heart failure: 207 (16.0%); stroke: 144 (11.2%); hypertension: 911 (70.6%)
	Endocrine system diseases	1102 (5.3%)	24 (21.2%)	N/A	Diabetes: 180 (17%); hypercholesterolemia: 188 (18%)	Type 2 diabetes: 409 (31.7%); obesity: 129 (10.0%)
	Digestive system diseases	N/A	1 (0.9%)	N/A	N/A	N/A
	Respiratory system diseases	511 (2.4%)	11 (9.7%)	N/A	COPD^: 42 (4%)	COPD 234 (18.1%)
	Chronic hepatitis B virus infection	N/A	5 (4.4%)	N/A	Chronic liver disease: 28 (3%)	Chronic liver disease: 49 (3.8%)
	Chronic kidney disease	N/A	4(3.5%)	N/A	36 (3%)	298 (23.1%)
	Malignant tumor	107 (0.5%)	1 (0.4%)	N/A	81 (8%)	217 (16.8%)
	Dementia	N/A	N/A	N/A	N/A	203 (15.7%)
	Autoimmune diseases	N/A	1 (0.4%)	N/A	N/A	40 (3.1%)
	*Median days from symptom onset to date of diagnosis (median, interquartile range)*	N/A	10 (7–13)	5.1 (95% confidential intervals, 4.5–5.8)^f^	N/A	N/A

*N/A* not available.

^a^See ref. ^12^ (A summary of a report of 72314 cases from the Chinese Center for Disease Control and Prevention).

^b^See ref. ^90^.

^c^See ref. https://www.epicentro.iss.it/coronavirus/bollettino/Bollettino-sorveglianza-integrata-COVID-19_6-aprile-2020.pdf.

^d^See ref. ^91^.

^e^See ref. https://www.epicentro.iss.it/coronavirus/bollettino/Report-COVID-2019_6_aprile.pdf

^f^Absolute number not available.

Lymphopenia and leukopenia has been observed in the majority of patients, together with elevated level of C-reactive protein, lactate dehydrogenase, D-Dimers, and other inflammatory biomarkers, including tumor necrosis factor-α (TNFα), interleukin-1β (IL-1β), IL-6, granulocyte-macrophage colony-stimulating factor as well as IL-10 (refs. ^1,8–11^). T cell exhaustion, especially CD4+ T cells is a hall marker of infected patients, paralleling with the severity of the illness^8^.

The vast majority only suffered from mild symptoms. However, the early report in Chinese involving 44,672 confirmed cases, 81% patients are mild cases, and 14.8% patients are severe, while only 5% patients are critically ill^12,13^. Numbers of comorbidities were associated with poorer outcomes^14^. The median onset time from early symptoms to dyspnea is around 7 days, while acute respiratory distress syndrome (ARDS) developed around 9 days^1^. The median days of fever in survivals is 10–12 days and cough persisted for 19 days^8,10^. The severity of the diseases varies in different age groups, with older patients at higher risk of mortality compared to those of younger age. In children, the symptoms are often mild and the prognosis of pediatric patients is largely more favorable than adults^15^. Table 1 compares the major characteristics of COVID-19 in China versus Italy. Patients in Italy were more older compared to patients in China, with more numbers of comorbidities. The number and severity of these co-morbidity has been a major factor influencing the outcome; this was particularly evident where the virus diffused into old pension homes. Indeed, while the mortality was 3.1% in Italy, with the exception of the Milan area (Fig. 1b) where it was 6.8%, within the residences for old people it peaked to 24% (https://www.epicentro.iss.it/). In this case, out of 3859 death, only 133 were confirmed by swabs, while 1310 had all symptoms but they were not tested. Disease severity was strongly age dependent, primarily due to the presence of comorbidities. Therefore, the proportions of severe and critical patients in Italy were higher than that in China, partially leading to a higher mortality.

### Asymptomatic patients

A proportion of the patients showed no symptoms at enrollment as they were at very early stage of the diseases. These patients could either recover without developing symptom or would continue to develop symptoms. However, the former group of patients never have any symptoms or signs, but their respiratory tract specimens are PCR positive for the virus. The exact number of the proportion of asymptomatic patients requires longitudinal study with repeated PCR tests. In a study that followed 13 patients in Wuhan, China, 31% of them never developed symptoms^16^. In another study performed on the Diamond Princess cruise ship, repeated PCR testing of 3711 quarantined passengers and crew members showed that asymptomatic proportion is around 18%^17^. More recently, the proportion of infected people have mild or asymptomatic were estimated to represent some 60% of all infections^18^. Notably, asymptomatic and symptomatic patients show comparable viral load, suggesting that these patients have strong transmission potentials^19^. Indeed, viral transmission from asymptomatic carriers have been reported^20^. In a recent study from China, Chen et al. followed up 2147 close contactors of 191 patients (161 symptomatic and 30 asymptomatic). They found that the infection rates of transmission rate in symptomatic cases was 6.3% comparing with 4.1% in asymptomatic patients, indicating the importance of identification and isolation of asymptomatic patients in the effort of containment the spread of the virus^21^. Importantly, for large-scale screening, antibody testing should be combined with PCR to avoid asymptomatic viral spreading.

## Diagnosis of COVID-19

### Nucleic acid tests

The definitive diagnosis of the disease relies on the identification of viral genomic RNA using either PCR-based technology or deep sequencing. The detailed PCR-based methods have been reviewed elsewhere^22^. The presence of sufficient viral genome for amplification at the site of sample collection is the precondition of the tests. Therefore, collecting the appropriate specimen from patients at the right time using the adequate protocol is a key in diagnosis of the infection. Besides in the respiratory tract, viral particles have also been detected in the blood, lacrimal fluid, urine, and feces^23,24^. In a recent study, the virus was detected in different types of samples, such as bronchoalveolar lavage fluid (BALF) (93% positive), pharyngeal swabs (32%), fibrobronchoscope brush (46%), nasal swabs (63%), feces (29%), sputum (72%), and blood (1%)^24^. However, the samples were not from the same patients at the same time, making it impossible to compare the sensitivity of the PCR test in different types of specimens.

As a viral pneumonia, respiratory tract specimens from COVID-19 patients are first of choice to collect for the detection of viral nuclear acid. Collecting and testing upper respiratory specimens would be ideal considering the feasibility in clinical practice. Nasopharyngeal and oropharyngeal swab are the optimal manner for swab viral testing. These samples can even be collected by patients themselves. Self-collected saliva specimens had been test and yield positive results in most of the infected patients, indicating that it is an adequate non-invasive test for monitoring and diagnosis of the infections^25^. To increase the sensitivity of the diagnosis, it is preferably to collect multiple different types of specimens. However, negative naso-oro-pharyngeal swab could not completely rule out COVID-9 (ref. ^26^). Cases with repeated negative PCR for the virus in naso-oro-pharyngeal tests but positive in bronchoalveolar lavage fluid (BALF) samples have been reported^26^. It is therefore necessary to collect BALF via bronchoscopy in patients highly suspected of COVID-19, although this procedure could significantly increase the safety danger to healthcare personnel through the creation of aerosol droplets. This is an important issue, considering that in Italy alone, over 170 doctors have died so far!

As soon as possible on the earliest days of symptoms, swabs should be collected from the upper respiratory tract. Recent studies showed that the viral load in the upper airway samples peaks within the first week and the virus was cleared at a median of 9 days from infection^25–27^. Similar shedding patterns were seen for MERS-CoV and SARS-CoV: RNA swab positivity was evident within the first week of MERS infection, while it peaked at 7–10 days after symptom onset in SARS. The dynamic of SARS-CoV-2 shedding in the lower respiratory tract specimens is still unclear. In MERS, MERS-CoV viral load peaked between week 2 and 3 in lower airways specimens, while the SARS-CoV RNA-positive rates in lower airways samples remained higher for 3 weeks after beginning of illness in SARS^28,29^. It is likely that SARS-CoV-2 also shares a comparable viral shedding pattern in the lower respiratory tract specimens. This may explain why some patients progressed despite PCR turned negative in the upper respiratory specimens^8^.

Despite the high accuracy of the PCR tests, these nucleic acid tests highly depend on the procedures of sample collecting, in addition to high labor and time costing. The test protocol is generally expensive and complex, and therefore difficult to be broadly applied in resource limited locations. Transferring samples to a central laboratory not only decrease the accuracy of the tests either by sample decay due to incorrect storage or contamination but also further delay the test results. A false-positive test could result in an unnecessary quarantine and therapy while a false-negative diagnosis may allow infected patients to spread the virus. Therefore, other methods are also needed to serve as a supplement. Most importantly, as the virus spreading many healthcare personnel have caused wanton community transmission before correct testing allowed isolation and tracking. Therefore, tests that are able to be scaled up quickly are urgently needed.

### Serological tests

Serological assays detect both SARS-CoV-2-specific antibodies IgM, IgA, and IgG, which are produced both at the early and later phases of the disease, respectively. Unlike PCR tests, antibody tests could be less accurate and require longer time to be established as routine tests. However, serological tests are portable, user-friendly, and can produce results in 10–30 min at least at the qualitative level. Moreover, they request very limited sample volume: one drop of blood from a pinprick is sufficient to detect the antibodies. Therefore, they can be established as decentralized point-of-care (POC) tests. In a cohort comprised of 82 confirmed and 58 probable (qPCR test is negative despite other indications of COVID-19 including symptoms and epidemiology) COVID-19 cases, the positive detection rate is extremely high (98.6%) when PCR is performed in conjunction with IgM ELISA for every patient as compared to qPCR analysis alone (51.9%)^30^. Therefore, the serological tests could be used together with nuclei acid detection to increase the sensitivity of tests. In China, some of these kits have received fast-track approval from the National Medical Products Administration (NMPA). In the USA, only one serological test has been approved by the date of submission. These tests are of good accuracy with sensitivity range from 87.3% to 97.2% and specificity range from 95.6% to 100%. The detailed serological tests has been reviewed elsewhere^31,32^.

However, the sensitivity of serological assays is influenced by both the time of sample collection as well as human immune response status. In the study by Guo et al., as high as 22.0% (18/82) of the PCR confirmed cases were found to be negative by an IgM antibody tests. Most of the COVID-19 patients were able to generate antibody within 10 days after symptoms onset. Suspected patients who tested IgM/IgG negative in their early phase of disease could have repeated IgM/IgG tests^30^, if nucleic acid tests are not available. In addition, the serological assays can also provide historic information about viral exposure, which are important for epidemiology studies. Interestingly, early presence of IgA might be protective and a surrogate marker of good outcome, detecting of which may help identifying patients warrant medical attention^33,34^. Therefore, the speed and versatility of serological assays make them invaluable tools for COVID-19 diagnosis and efforts to produce antibody detection kits on a huge scale have begun to be produced^31^. Clearly, serological determination is the future both for diagnostic as well for epidemiological purposed.

### Computer tomography

**Fig. 5:**
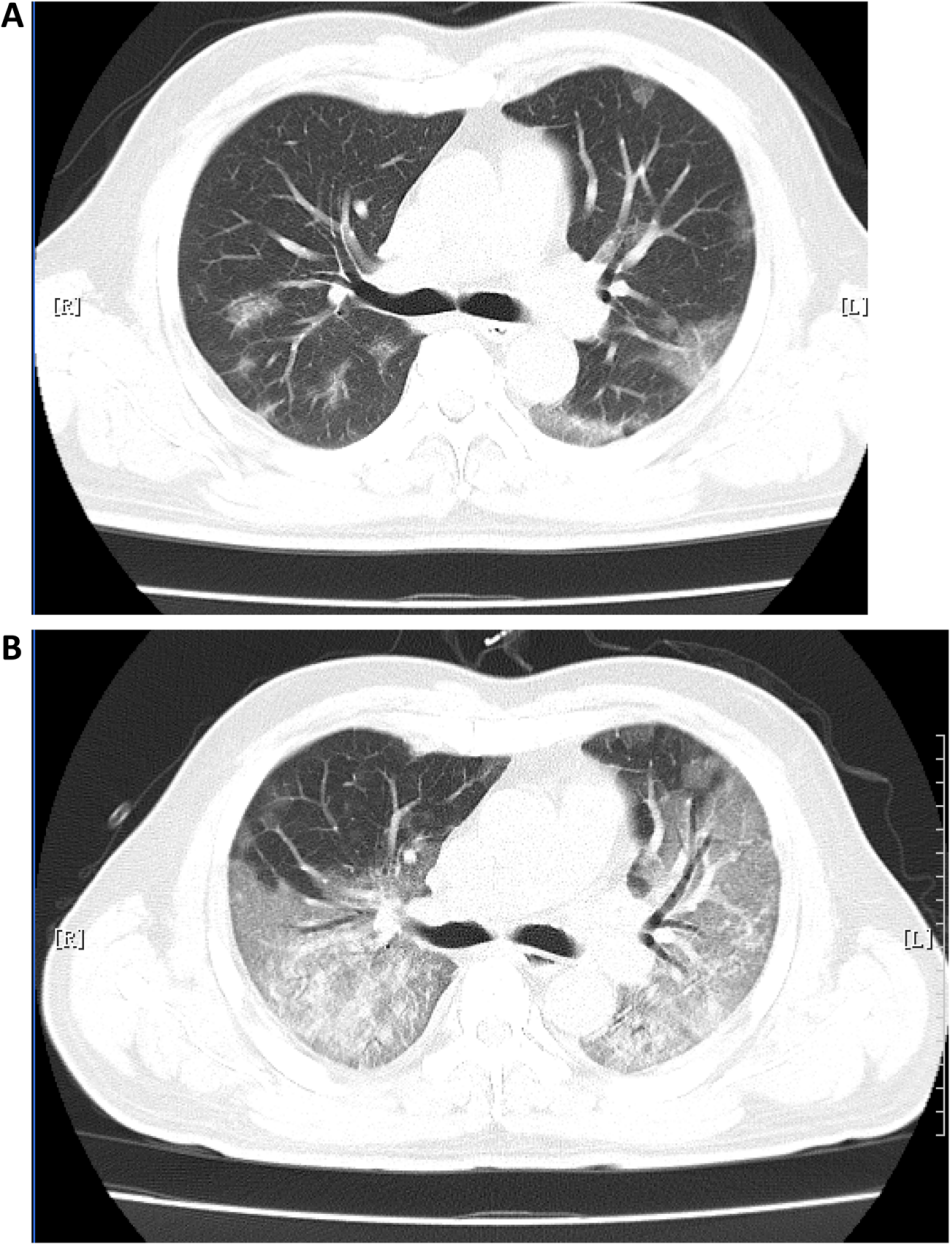
Computer tomography image of COVID-19. Early (**a**) and late stage of COVID-19 (**b**) computer tomography of the lung showing a diffuse interstitial pneumonia.

The COVID-19 patients presented some typical characteristics on the chest computed tomography (CT) image. Ground-glass opacity was the most frequent, with subsequent consolidations, air bronchogram, irregular or smooth interlobular septal thickening, and thickening of the nearby pleura, with predominantly lower and peripheral lobe involvement^9,35^ (Fig. 5). A recent study by Ai et al.^36^ involved 1014 patients in China reported that the sensitivity of toracic CT in suggesting COVID-19 was 97%. In the early February, 2020, some experts in China suggested CT as one of the best clinical diagnostic criteria together with epidemiology, clinical, and laboratory characteristics to pronounce the clinically diagnosed COVID-19 when PCR tests were not available. Nonetheless, not all the patients had the typical chest CT images (e.g., asymptomatic COVID-19 cases). Absence of CT or radiographic abnormality was observed in as high as 17.9% patients with non-severe disease and in 2.9% cases with severe disease^9^. Notably, the typical CT image sometimes could not distinguish COVID-19 from influenza infections. In the study by Ai et al.^36^, 75% of patients with negative RT-PCR swabs showed positive thoracic CT results. Given that the misdiagnosis of COVID-19 in cases has grave consequences as mentioned above, CT should not be used alone to make the diagnosis of COVID-19.

## Clinical management

At present, there are no specific treatment with confirmed efficacy for COVID-19 and the therapeutic protocol is as per best supportive care for any respiratory disease. However, both antivirals and immune modulators warrant further investigation especially considering the biphasic pattern of immune responses during the diseases course^8^. Several drugs with potential antiviral activities are under active investigation. More recently, the WHO also launched multinational trial to validate four drugs especially in combinations, including ritonavir/lopinavir, lopinavir and ritonavir with interferon-beta, chloroquine and/or remdesivir. Several clinical trials, over 1673, have been registered worldwide and are listed at www.clinicaltrials.gov (specifically see: https://clinicaltrials.gov/ct2/results?cond=covid-19). A selected clinical trials with possible significance are listed in Table 2.

**Table 2:** A selected examples of significant clinical trials.

	Conditions	Numbers of enrollment (estimated)	Results	Reference/trials
Hydroxychloroquine	Mild	30	Negative	^39^
Hydroxychloroquine	Mild/severe	62	Positive	^41^
Hydroxychloroquine	Symptomatic disease	510	Ongoing	https://clinicaltrials.gov/ct2/show/NCT04332991
Hydroxychloroquine	Prevention	40,000	Ongoing	https://clinicaltrials.gov/ct2/show/NCT04303507
Lopinavir/ritonavir	Severe	199	Negative	^51^
Remdesivir	Mild/Moderate	308	Ongoing	https://clinicaltrials.gov/ct2/show/NCT04252664
Remdesivir	Severe	453	Negative	^59^
Remdesivir	Severe	2400	Ongoing	https://clinicaltrials.gov/ct2/show/NCT04292899
Remdesivir	Moderate	1600	Ongoing	https://clinicaltrials.gov/ct2/show/NCT04292730
Remdesivir	Moderate/Severe	800	Ongoing	https://clinicaltrials.gov/ct2/show/NCT04280705
Dexamethasone	Severe	200	Ongoing	https://clinicaltrials.gov/ct2/show/NCT04325061
Tocilizumab(IL-6 inhibitor)	Severe	20	Positive	^84^
Tocilizumab	Mild/Severe	188	Ongoing	http://www.chictr.org.cn/showprojen.aspx?proj=49409
Anakinra (IL-1 inhibitor)	Mild/Severe	342	Ongoing	https://clinicaltrials.gov/ct2/show/NCT04330638
Baricitinib (JAK inhibitor)	Mild	80	Ongoing	https://clinicaltrials.gov/ct2/show/NCT04340232
Convalescent plasma	Severe	10	Positive	^63^

### Chloroquine and hydroxychloroquine

Chloroquine (CQ) and its related drug hydroxychloroquine (HCQ), both of which are well-known for their effectiveness in treating malarial and autoimmune diseases, have also recently been repurposed to treat COVID-19. In vitro studies showed that both CQ and HCQ were of excellent antiviral activity against SAR-CoV-2 with an EC_50_ of 1.13 and 0.72 μM, respectively, which are reachable in humans when admitted^37,38^. In fact, CQ and HCQ have been shown to have antiviral activities to different viruses including dengue virus, chikungunya virus, Ebola virus, etc. Any effect found has been limited to in vitro culture. There is no in vivo efficacy reported. Many randomized controlled trials are undergoing to evaluate the efficacy of CQ and HCQ in treating or even in preventing COVID-19. We completed the first randomized, control, open-label trial to evaluate the antiviral activity and safety of HCQ in treating COVID-19. Unfortunately, in this pilot study with 30 patients, we failed to observe any benefit of adding HCQ on top of the standard of care^39^. However, more recently, another study from France showed that HCQ was effective against SARS-CoV-2, especially when combined with azithromycin^40^. These two studies were both limited by the small sample size. The U.S. Food and Drug Administration (FDA) has launched a trial to investigate the effectiveness of CQ in COVID-19 (https://www.fda.gov/news-events/press-announcements/coronavirus-covid-19-update-fda-continues-facilitate-development-treatments). More recently, a study was completed in Renmin Hospital of Wuhan University with 62 COVID-19 patients^41^. This study was based on a follow-up survey, showing that none of the 80 SLE patients who took long-term oral HCQ showed SARS-CoV-2 infection or appeared to have related symptoms. The study found that the cough remission time and the body temperature recovery time were strongly reduced in the HCQ treatment group. Over 80% (25 of 31) of the patients in HCQ treatment group showed improvement in pneumonia compared with the matched controls (54.8%, 17 of 31). Importantly, 61.3% of patients in the HCQ treated group showed significant pneumonia absorption. Among the 31 patients in the HCQ treated group, 2 of them had mild adverse reactions, one developed rash and one patient experienced a headache. Clearly, these results warrant further randomized, controlled studies. Remarkably, HCQ is a strong immune modulator and has been used widely for the therapy of systemic lupus erythematosus or rheumatoid arthritis. It exerts its effects likely through inhibiting the secretion of IL-1β, TNF-α, and IL-6 from macrophages/monocytes, which are a key cell population in ARDS development in COVID-19 patients. Thus HCQ application should be limited to patients who have started to develop or already developed ARDS. Since immune response at the early stage is critical for controlling infection and the antiviral effect of HCQ is still in question. In addition, the safety of CQ/HCQ should also be taken into consideration. CQ/HCQ are associated with cardiac arrhythmias, hypoglycemia, and neuropsychiatric effects^42^. Therefore, these compounds should not be recommended for mild patients or for prevention purpose, at least for now.

Moreover, the combination of HCQ with antibiotic drug azithromycin has been reported to have added benefit. Since azithromycin is known to reversibly binds to the 50S ribosomal subunit of the 70S ribosome to inhibit the translocation step of protein synthesis, whether it has antiviral effect is not known. It is also not known whether azithromycin synergize with HCQ to provide better antiviral activity. A recent prospective study failed to find antiviral activity or clinical benefit of this combination for the treatment of our hospitalized patients with severe COVID-19 (ref. ^43^). Further investigations are needed. Needless to say, at late stage of ARDS, controlling secondary bacterial infection should not take lightly.

In addition to treatment, CQ/HCQ has also been suggested for prophylaxis of COVID-19 (ref. ^44^). When awaiting more evidence from clinical trials, we currently do not recommend use CQ/HCQ for prophylaxis of COVID-19.

### Lopinavir/ritonavir

Lopinavir/ritonavir (LPV/r) is a mix of protease inhibitors commonly used for the therapy of HIV-1 infection, often known as highly active anti-retroviral therapy (HAART). The combination of LPV/r with additional ribavirin was reported to be able to reduce the risk of acute respiratory distress syndrome and mortality in SARS patients^45^. Meanwhile, LPV/r and interferon β were found to have better efficacy compared with controls for MERS-CoV infection in animal experiments and case reports^46–49^. LPV/r was therefore repurposed to treat COVID-19, as SARS-CoV-2 was genetically close to MERS-CoV and SARS-CoV. However, in a retrospective study with 134 patients (more than 95% patients were mild cases), we recently find that administration of LPV/r along with interferon alpha inhaling was not associated with faster virological clearance or clinical improvement^50^. More recently, a randomized, controlled study conducted in Wuhan, China also failed to identify beneficial effect of LPV/r beyond standard therapy in hospitalized patients with severe Covid-19 (ref. ^51^). More importantly, in another randomized control study with a small sample size, LPV/r was associated with adverse events not only failed to show antiviral activity^52^. Based on these available data, it is likely that LPV/r and other HIV-1 protease inhibitors including darunavir are not effective in treating COVID-19.

### Remdesivir

Another promising drug is the adenosine analog remdesivir (GS-5734) that is incorporated into nascent viral RNA chains where it causes a pre-mature termination^53^. Remdesivir is also an experimental drug that was generated for the therapy of the Ebola viral infection. Notably, remdesivir has shown antiviral efficacy in treating SARS and MERS in animal models^54,55^. Recently, in vitro study showed that, in Vero E6 cells, EC_90_ value of remdesivir versus SARS-CoV-2 was 1.76 μM, indicating its active concentration could likely be obtained in vivo^37^. In SARS-CoV-2-infected rhesus macaques, therapeutic remdesivir treatment was found to reduce viral load when given early^56^. In the USA, the first COVID-19 patient was treated with intravenous remdesivir and recovered^57^. In a recent cohort of hospitalized patients with severe COVID-19, compassionate-use remdesivir was associated with clinical improvement in 36 of 53 patients (68%)^58^. In a randomized, double-blind, placebo-controlled, multicentre trial, remdesivir use was not associated with a difference in time to clinical improvement. However, when given with symptom duration of 10 days or less, patients receiving remdesivir had a numerically faster time to clinical improvement than those receiving placebo^59^. This study was underpowered as it failed to enroll the estimated number of participants. However, preliminary results from another NIH clinical trial shows remdesivir was associated with 31% faster time to recovery from advanced COVID-19 and a marginal survival benefit, with a mortality rate of 8.0% for the group receiving remdesivir versus 11.6% for the placebo group (*p* = 0.059)^60^. As an emergent therapeutic approach, FDA recently issued emergency-use authorization for remdesivir to treat hospitalized patients with severe Covid-19. Several clinical trials are currently ongoing, and the results regarding effectiveness and safety are still being awaited for.

### Camostat

The recent work elucidating the structure and molecular mechanism of viral entry^4–6^ showed the specificity of the serine protease inhibitor camostat mesylate as an active compond against TMPRSS2 which is required for viral infection. Camostat mesylate seems rather specific, since SARS-Cov-2 infection requires TMPRSS2 for the priming of the viral S protein. Clinical trial of camostat mesylate or its derivatives are therefore required^4–6^.

### Ivermectin

The recent work on the FDA-approved anti-parasitic compound ivermectin seems extremely powerful even though so far it is only in vitro^61^. Clinical trial to repurpose this approved drug is urgently needed.

### Convalescent plasma

Convalescent plasma is also a potentially promising strategy to treat COVID-19. In a recent case study, the clinical status of all the five critically ill COVID-19 patients receiving convalescent plasma showed a significant improvement within 1 week following the infusion, normalization of body temperature, as well as scores of the sequential organ failure assessment. Moreover, within 1–12 days following the infusion, the neutralizing antibody titers of the patients improved and the respiratory samples tested negative for SARS-CoV-2 (ref. ^62^). In another study of 10 severe cases, the viral titers were undetectable following the infusion in seven patients who had previously high viremia^63^. Previous studies on other respiratory viral diseases provided some evidences on the efficacy of convalescent plasma on treating severe and critical viral diseases. Several studies in SARS patients reported that the use of convalescent plasma was linked to reduced hospital stay and reduced mortality^64,65^. Clinical trials also showed that in patients with severe H1N1 influenza A, in the 2009 pandemic, therapy with convalescent plasma from patients who recovered, especially within 5 days of symptom onset, resulted in a lower viral load and lower mortality^66,67^. Subsequent analysis showed that the mortality of patients with severe acute viral respiratory infections was reduced after therapy with convalescent plasma, while absence of adverse events or complications were observed^68^.

Nevertheless, there are still issues we need to tackle. The first question is when to collect plasma from recovered COVID-19 patients. Recent work by To et al.^25^ showed that, day 10 after symptom onset, both IgG and IgM antibodies increased in the majority of patients, while seroconversion was observed within the first 3 weeks. Importantly, the anti-SARS-CoV-2 IgG and IgM antibody levels against the internal nucleoprotein and the spike S1 domain correlated with neutralizing activity. Therefore, it would be ideally to collect convalescent plasma from week 3 after symptom onset. Despite hundreds of patients had recovered from COVID-19, eligible convalescent plasma is quite limited as the donors have to pass physical and laboratory examination, and plasma should also be tested for SARS-CoV-2 nuclear acid, HIV-1, HBV, and HCV, as well as antibody titers, to list a few. The second question is deciding which patients and when should receive the convalescent plasma. The effects of convalescent plasma are difficult to observed when used in critical patients with multiple organ failure, as the viral load in this kind of population is quite high. Hence it is preferably to use convalescent plasma in mild patients whose diseases was deteriorating in their early phase of diseases. Normally, in COVID-19, the viral load peaked at the first week of illness, and then slowly decline during the subsequent week^25^. Accordingly, in principle, the most effective to administer the convalescent plasma is at the early phases of the disease. The biggest challenge is that it is quite difficult to identify which patient will deteriorate in the early stage. Several risk factors including older age, male, multiple comorbidities, elevated IL-6, and elevation in D-dimer levels that are associated with bad outcomes may be used as surrogate markers^10^. Provided further studies demonstrate its efficacy in appropriately selected patients, the next step would be the production of humanized antibody at biotechnological level.

## Cytokine storm syndromes and its management

It is still not clear why some patients progressed while others recovered, which underlying biological marker would be of essential benefit for the management of the patients. Indeed, several reports are still in wide contrast. The duration from onset of symptoms to viral clearance is significantly longer in severe and critical ill SARS-CoV-2-infected patients compared with that in the mild cases^48^. Notably, elevated level of a bundle of pro-inflammatory cytokines was observed in severe and critical ill patients, which include interferon-γ inducible protein 10, interleukin (IL)−2, IL-7, IL-6, macrophage inflammatory protein 1-α, granulocyte-colony stimulating factor, tumor necrosis factor-α, and monocyte chemoattractant protein 1 (refs. ^1,11^). More importantly, in a recent retrospective, multicenter study conducted in Wuhan, China, increased plasma levels of ferritin and IL-6 were identified as predictors of fatality^69^. Therefore, it is reasonable to speculate that the persistence of SARS-CoV-2 induced excessive and abnormal non-effective response that leads to organ dysfunction^70,71^. The inflammation persists in some patients despite the viral clearance^8,72^. Taken together, these evidences support the importance of dampening the overly exuberant immune responses besides antiviral therapy in reducing the mortality^73^, as we discussed previously^74^.

The dual role of the immune system^74^ is crucial and still under investigation to clarify the strength and duration of the immune response both for the perspective of post-infection protection and re-infection, but also, and most relevant, for the efficacy of the future vaccine.

Many therapeutic drugs are now available to suppress immune response. Among these drugs, corticosteroid is the most widely used. However, the utility of corticosteroid in treating COVID-19 is still debating. Most guidelines on COVID-19 currently do not recommend application of corticosteroid as data are very limited. On the one hand, corticosteroid apparently delay clearance of MERS-CoV from respiratory tract and SARS-CoV from blood, respectively^75,76^. On the other hand, clinical studies reported that low or physiologic dose of corticosteroids treatment could have clinical benefits to earlier reversal of shock, shorter stay in ICU, and less mechanical ventilation although it did not reduce mortality caused by primary lung infections^77^. In patients with severe H1N1-illness, low dose of corticosteroids also lead to lower mortality^78^. Several clinical trials are now in progress to evaluate the benefit of corticosteroids in treating COVID-19. When waiting for the results, proper use of low-dose corticosteroids at the right time might has survival advantages for severe/critically ill patients with COVID-19^79^.

CQ and HCQ might also be able to modulate the over activated immune response. They alter the pH and disrupt autophagy and lysosomal activity, destabilize membrane, and disrupt signaling pathways and transcriptional activity, subsequently inhibiting antigen presentation, immune activation, MHC class II expression, reduced pro-inflammatory cytokines, and deregulation of co-stimulatory molecules^80^. However, whether its suppression on the immune response may lead to delay of SARS-CoV-2 clearance is still unclear. In some viral diseases including HIV infection, HCQ administration was associated with increased viral replication^81^.

Other therapeutic drugs that under investigation include the IL-1 inhibitor, IL-6 inhibitor, and JAK-STAT signal pathway inhibitors. Treatment with IL-1 blockade, in a phase 3 randomized controlled trial in sepsis patients with macrophage activation syndrome, resulted in a drastic improvement in the 28-day survival rate^82^. IL-6 inhibitor, which was used as a treatment for rheumatoid arthritis and CRS associated with CAR-T cell therapies for cancer, has now repurposed to treating COVID-19 in severe and critical patients as recommended by a Chinese government guideline^83^. Interestingly, preliminary results from the two available IL-6 inhibitors (tocilizumab and sarilumab) with different study population showed contrasting results, indicating that choose appropriate population is essential for anti-IL-6 treatment^84^. Upstream of that, the treatment with inhibitors of TNF-β could be applied, but there are no data available so far. Inhibition of Janus kinase (JAK) in COVID-19 patients target both inflammation and cellular viral entry^85^. Recently, a pilot study suggest that JAK inhibitor is active, well tolerated in patients with secondary hemophagocytic lymphohistiocytosis indicating these drug maybe apply to COVID-19 treatment^86^.

## Discharge and follow-up

Most of the patients can be discharged after archive negative PCR results from two continues respiratory tract specimens, together with defervescence and improvement in radiological image^83^. The median duration from symptoms onset to the first negative PCR results was 12 days in non-intensive care units group, as compared with 26 days in the ICU groups. In critical ill patients, the time of viral shedding can be as long as 52 days. The slower clearance of the virus in the critical ill patients might be explained by both higher viral load at baseline and impaired immune responses. In a recent study, the mean SARS-CoV-2 viral load of critical ill patients was circa 60 times higher than that of moderate cases, indicating that higher viral loads is linked to severe clinical outcomes^87^. In addition, the critical ill patients also showed impaired immunity characterized by lymphopenia and lower CD4 T cell counts, which were associated with the duration of viral shedding^8,27^. Our cohort in Shanghai showed the median duration of hospital stay was 16 days^8,12,20^. However, studies have shown that viral shedding is longer in the feces than in the respiratory tract specimens^23^. More importantly, SARS-CoV-2 isolated from feces was of replicability. Despite the uncertainty of transmissibility of SARS-CoV-2 in feces, it indeed challenged the quarantine given the high infectivity of the virus. Notably, PCR turned to be positive again in a small proportion of patients, although no infection cases of these close contactors had been reported. Therefore, we recommend that patients recovered and discharged should be continue to quarantine for another 14 days.

## Preventive measures to minimize recurrence of epidemics

**Fig. 6:**
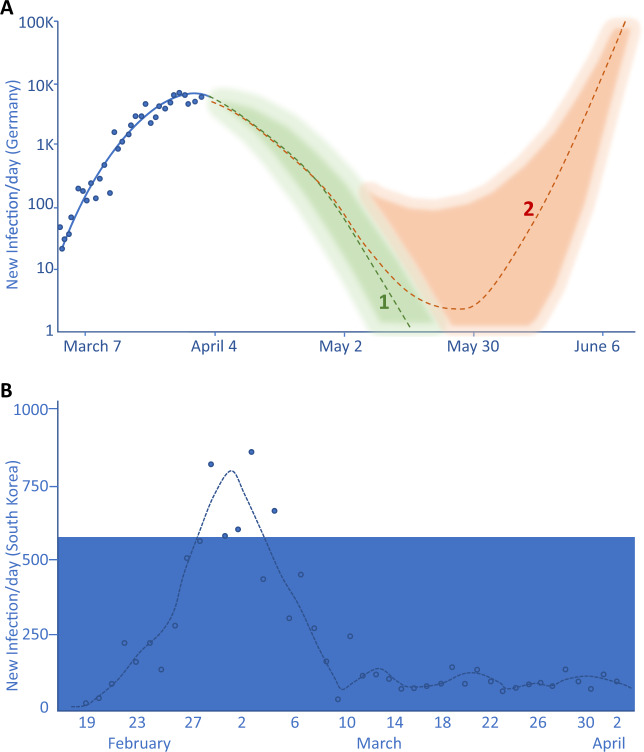
Daily incidence of new infections and future scenarios. The Leopoldina Statement reported the daily incidence of new covid-19 infections in Germany (**a** blue dots and line) followed by a statistical modeling of new infections, with full implementation of all proposed measure of containment (hygiene recommendations, physical distancing, nose and mouth protection, testing, digital data tracking, selected targeted quarantine) with a very gradual relaxation of restrictive measures imposed to public life (**a**, green line 1). Conversely, the scenario proposes a fast reactivation of the infections (**a**, reddish line 2) if the proposed measures fail to be implemented. The read frequency of cases in South Korea (**b**), despite full implementation, shows that sporatic new infections cannot be fully avoided despite serious prevention, resulting in a low grade of endemic cases^88^.

Some models had attempted to quantify the impact of different strategies to prevent the potential spread of COVID-19 that should be carefully considered for policy makers in planning the next steps. In interpreting these models, we should keep in mind that they assume that variations in the replication rate of SARS-CoV-2 - an evaluation of transmission - are an immediate response to the interventions implemented, rather than broader gradual behavioral changes. While first models, however, addressed a set of variables before locking down, on the number of cases and avoided deaths, more recently new models are struggling to predict the effect of gradual normalization of public life of different strategies. In fact, despite the fact that in Italy, Spain, and Germany the increase in number of new SARS-CoV-2 infections has been reducing in recent days, the political measures (on early April 2020) for restricting contacts at national level continue to be in place. In a recent report from the Leopoldina Nationale Akademia der Wissenchaften^88^, authors concluded that in addition to the current recommendations (hygiene, physical distancing), also nose and mouth protection, testing, use of digital data and targeted quarantine should be reinforced, see Fig. 6 and also ref. ^89^. Assuming, if optimally implemented, that the rate of new infections caused by an distinct person could be lower than 1.0, the aforementioned measures might strongly impact in reducing further spread of SARS-CoV-2, with a simultaneous gradual relaxation of restrictive measures imposed on public life^88^.

## Conclusion and way forward

After several months’ lockdown, China is now reopening, facing the risk of second wave of COVD-19. In Italy, despite lots of patients had already been infected, second wave of diseases is also challenging should they reopen the cities, as there is currently no evidence that people who have recovered are protected from a second infection. While waiting for an effective vaccine for COVID-19 and for a clear specific treatment, current management is highly depended on supportive therapy. The best way to reduce the mortality is therefore related first of all to risk reduction of SARS-CoV-2 infection. This might be achieved by combining six main actions: social distance, masks, hygiene recommendations, contact tracing, extensive use of early diagnosis tools, and confirmed and suspected cases quarantine. We learnt from countries like South Korea and Singapore that such approaches can be feasible, even in absence of very strict lockdown. Secondly, strengthen health systems capacity in order to be fully equipped in terms of medical staff and optimal equipment’s to prevent medical resource be overwhelmed. The implementation of these measures is pivotal to prevent re-infections and reactivation of the pandemic; therefore, the future working society and economy must adapt to these measures.
